# Eddowes and Others v. St. John's Hospital

**Published:** 1913-06-28

**Authors:** 


### EDDOWES AND OTHERS v. ST. JOHN'S HOSPITAL.

### The Tenure of Hospital Staff Appointments.

BY AN HONORARY MEDICAL OFFICER.

The important action brought by Drs. Eddowes, Dawson, and Knuthsen against St. John's Hospital for Diseases of the Skin terminated last week in favour of the plaintiffs, who were awarded £50 apiece by way of damages. But the injunction which they sought, to restrain the hospital authorities from excluding them from the hospital was not granted. To hospital administrators especially, and to members of the medical profession in a somewhat lesser degree, the issues which arose in this trial are of very great interest. Besides these issues there are others which are so germane to them, though they did not actually arise, that they may usefully be taken into consideration together. Indeed, reflection upon the facts of this lawsuit speedily suggests that the whole principle and practice of the selection and tenure of medical staffs in our voluntary hospitals is involved.

### The Facts of the Case.

Let us glance briefly first at the grounds of litigation between the parties of this present dispute. The plaintiffs were at one time members of the honorary medical staff of St. John's Hospital, and apparently were for some time at loggerheads with the senior physician. Ultimately they complained to the Board of some of the acts of this person, and a sub-board was appointed to consider the complaints. As a result of investigations, this sub-board, and on its recommendation the Board itself, decided in favour of the senior physician; and as a consequence of this decision it was also decided that the three members of the staff who had preferred the complaints would have to leave. In pursuance of this resolve, the Governors dismissed the three doctors without notice, and, as they contended, without justification.

Mr. Justice Pickford's summing-up, which presents the material facts with admirable terseness, fairness, and common-sense, shows that his decision (the case was tried without a jury) was based mainly on the summary and arbitrary method by which the Board of the hospital turned out the three doctors, rather than on anything inherently *ultra vires* in the decision to dismiss them. It was not necessary for him to decide, he said, whether the view that the medical staff, once appointed, were there for life (save in the case of incompetence or misconduct) is or is not correct; and he did not so decide. But he did express the opinion that honorary staff appointments must at least be terminable only on a proper notice; and he did not consider the seven days' notice which was mentioned as constituting proper notice.

### A Monstrous Contention.

It is clear from the Judge's remarks on this point that he does not uphold the contention put forward by some medical men that, in the absence of specific conditions limiting the duration of the appointment in some definite way, staff appointments in the voluntary hospitals are irrevocable and lifelong. This view of Mr. Justice Pickford's is, of course, merely a judicial *obiter dictum*, and not necessarily binding on other Courts; but it is so eminently reasonable that it is most unlikely that any tribunal will take the opposite view. To say that the governing body of a hospital have no power to dispense with the services of a member of the honorary medical staff, save for incompetence or misconduct, is, in our judgment, a monstrous proposition, as little in the interests of the medical profession as of the hospitals and their patients.

One further point the Judge did decide. He dissented from the suggestion put forward by counsel for the defence that the Governors had power to dismiss members of the staff simply on the ground of continued friction between them and other members of the staff. The making of complaints did not constitute, in the Judge's view, misconduct, such as would justify the summary dismissal.

### The Broader Issues at Stake.

So much for the facts as disclosed to Mr. Justice Pickford, and the decision taken upon them—another feather in the cap, by the way, of the London and Counties Medical Protection Society. In backing up their senior physician, the authorities of St. John's Hospital have run themselves into the inconveniences of an unsuccessful lawsuit, and are doubtless finding the luxury an expensive one. St. John's Hospital has had at various times thoroughly sound men upon its medical staff, the late Dr. Savill for example. But it would be quite idle to pretend that the staff as a whole has ever been first-rate; and in dermatological circles this hospital, which ought to command the best talent in London, has neither deserved nor obtained professional respect. So much will be conceded, we think, by anyone who is conversant with the general current of expert medical opinion in London.

Dermatology has in the past been so much mixed up with cosmetics and "beauty specialism" that an element of quackery and humbug tended to overshadow the proper development of the scientific side of this department of medicine. The younger generation of dermatologists are shaking off this reproach; but the taint still clings, and deters many young men of character and ability from taking up dermatology as a specialty. Quarrels and bickerings seem to chequer the annals of hospitals for diseases of the skin, in London at any rate, in much larger proportion than at other hospitals, as can be shown by anyone who cares to search back files of The Hospital. And this propensity is probably due to the fact that the general level of ability, courtesy, and broadmindedness is less high among the medical staffs at skin hospitals than at, let us say, the general hospitals. The best London dermatologists, professionally as well as socially, are those attached to the special skin departments of the general hospitals, not those on the staff of the special hospitals; probably this is the only recognised specialty in which such a statement would be accurate.

The Tenure of Staff Appointments.

The development of the voluntary system to its present stage has been rendered possible only by the unstinted devotion, through several generations, of the medical profession, and especially of that section of the profession from which the visiting staffs are recruited. When a man has thus laboured without reward, and given of his best work and energy to a charitable institution, naturally enough there is a disinclination to suggest or to enforce his resignation on account of his past services. Thus has grown up gradually the habit of regarding all members of visiting medical staffs as permanent officers, irremovable except for gross misconduct, in a professional or some other respect.

The custom, which although general was certainly never universal, has in the past often been the source of impaired efficiency and even worse in hospital affairs. So much has this been realised that in recent years several hospitals have adopted specific rules under their charters of incorporation limiting the duration of staff appointments to five years, renewable at the option of the Governors for similar periods. The majority of hospitals have not yet got as far as this; but an age-limit at which members of the medical staff must retire from it has been adopted at very nearly all hospitals. As a rule, this limit is sixty-five years for physicians and sixty years for surgeons.

For our own part, we regard the system of quinquennial re-election as much the best safeguard so far devised to protect institutions against the chance of being saddled with undesirable or ineffective medical men on their visiting staffs. We could quote plenty of instances where such a rule would certainly have been put into operation, had it existed, to the great benefit of hospitals and patients alike. The method of selection and election of new members of the medical staff works, on the whole, very well. But it is not infallible, and mistakes thus made should be rectified at convenient intervals afterwards.

